# Long-Term Increase in Cholesterol Is Associated With Better Cognitive Function: Evidence From a Longitudinal Study

**DOI:** 10.3389/fnagi.2021.691423

**Published:** 2021-06-17

**Authors:** Huamin Liu, Lianwu Zou, Rui Zhou, Minyi Zhang, Shanyuan Gu, Jiazhen Zheng, Daniel Nyarko Hukportie, Keyi Wu, Zhiwei Huang, Zelin Yuan, Xianbo Wu

**Affiliations:** ^1^Department of Epidemiology, School of Public Health, Southern Medical University, Guangzhou, China; ^2^Department of Psychiatry, Baiyun Psychiatric Rehabilitation Hospital, Guangzhou, China; ^3^Inpatient Department, Baiyun Psychiatric Rehabilitation Hospital, Guangzhou, China

**Keywords:** cognitive decline, episodic memory, mental intactness, cholesterol, longitudinal study

## Abstract

**Background:** Higher visit-to-visit cholesterol has been associated with cognitive decline. However, the association between long-term increase or decrease in cholesterol and cognitive decline remains unclear.

**Methods:** A total of 4,915 participants aged ≥45 years with normal cognition in baseline were included. The participants were divided into four groups, namely low–low, low–high, high–low, and high–high, according to the diagnostic thresholds of total cholesterol (TC), non-high-density lipoprotein cholesterol (NHDL-C), low-density lipoprotein cholesterol, and high-density lipoprotein cholesterol (HDL-C) after 4 years of follow-up. Cognitive function was assessed by episodic memory and mental intactness. Binary logistic regression was used to analyse the association of cholesterol variation with cognitive decline.

**Results:** Among the participants, 979 (19.9%) experienced global cognitive decline. The odds ratio (OR) of global cognitive and memory function decline were remarkably lower in participants in the low–high NHDL-C group than those in the low–low group [OR and 95% confidence interval (CI): 0.50 [0.26–0.95] for global cognitive decline, 0.45 [0.25–0.82] for memory function decline]. The lower OR was also significant in females (OR [95% CI]: 0.38 [0.17–0.87] for global cognitive decline; 0.44 [0.19–0.97] for memory function decline) and participants without cardiovascular disease (OR [95% CI]: 0.31 [0.11–0.87] for global cognitive decline; 0.34 [0.14–0.83] for memory function decline). The increases in other cholesterol were also negatively associated with the risk of cognitive decline although not significantly.

**Conclusions:** A longitudinal increase in NHDL-C may be protective for cognition in females or individuals without cardiovascular disease.

## Introduction

Cognitive decline is one of the greatest causes of disability (Lee et al., 2018). It is also a critical period for the prevention of neurodegenerative diseases, such as dementia and amyotrophic lateral sclerosis (Anderson, 2019). However, cognitive decline has always been a primary public health issue globally. Approximately 30% of Americans and 20% of Chinese aged over 65 years have cognitive decline, and the prevalence of cognitive decline is gradually increasing worldwide (Alzheimer's Association, 2016; Li et al., 2020). Thus, it is imperative to prevent cognitive decline.

Cholesterol is a well-established risk factor of cardiovascular disease that further promotes cognitive decline. Higher total cholesterol (TC) or low-density lipoprotein cholesterol (LDL-C) is associated with worse cognitive function (Stough et al., 2019; McFarlane et al., 2020). However, current studies do not yield consistent results. In the Longitudinal Aging Study Amsterdam, a lower TC level is associated with worse general cognition and information processing speed (van den Kommer et al., 2009). Chinese studies find that a high LDL-C level is associated with lower risks of dementia and cognitive decline, but the protective effect of high-density lipoprotein cholesterol (HDL-C) is only observed in women (Lv et al., 2016; Zhou et al., 2018). The gender-specific association is also validated in a French study, in which higher TC and LDL-C and lower HDL-C are associated with an increased risk of cognitive decline in French men but not women (Ancelin et al., 2014). These discrepant results add to the complexity of developing a prevention strategy for cognitive decline.

Long-term variation in cholesterol has become an interesting indicator for cognitive decline in recent years. Previous literature has documented that higher visit-to-visit cholesterol variation, an intraindividual variation index measured by the coefficient of variation (CV), variability independent of the mean (VIM), or standard deviation (SD), is correlated with worse cognitive performance regardless of baseline cholesterol levels (Smit et al., 2016; Lee et al., 2018). However, high intraindividual variation may be observed in the two variation patterns of cholesterol determined by diagnostic threshold: from low to high and high to low levels. Little is known about which variation direction of cholesterol is associated with cognitive decline.

The purpose of this study was to investigate whether the long-term variation in cholesterol from low to high or high to low levels is associated with cognitive decline. The variation patterns of cholesterol consist of a persistent low level, from low to high levels, from high to low levels, and a persistent high level based on two blood tests in 2011 and 2015. Given that cholesterol has a gender-specific association with cognition and a close association with cardiovascular disease, we also conducted subgroup analyses according to sex and the presence or absence of cardiovascular disease.

## Materials and Methods

### Study Population

**Figure 1:**
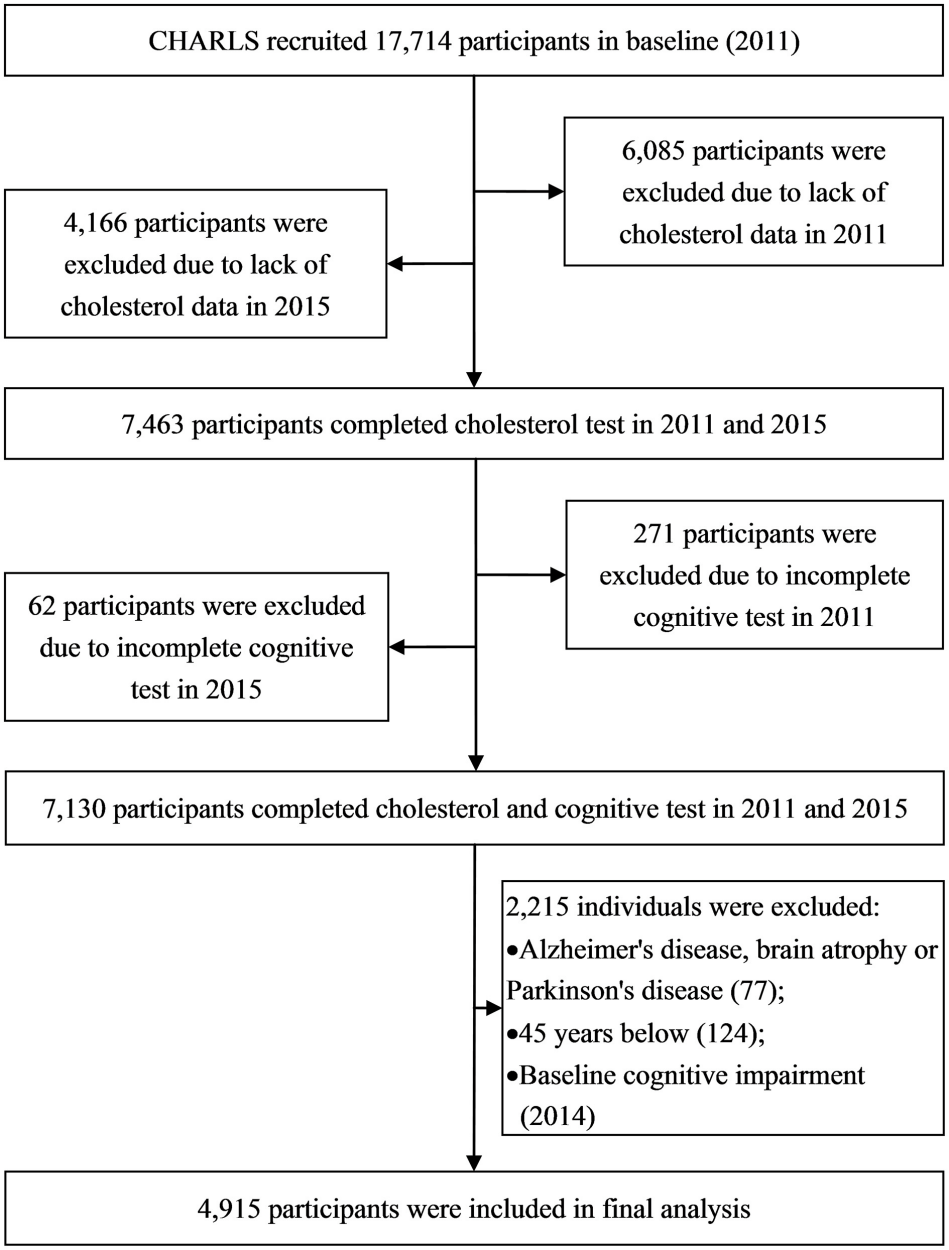
Flowchart of participant selection.

The China Health and Retirement Longitudinal Study (CHARLS) is a nationally representative survey initiated in 2011 that aimed to collect health-related information among Chinese who are over 45 years old and their spouses. The investigation areas consist of 30 provinces in which 450 villages/urban areas and 10,287 households were randomly selected by probabilities proportional to size. A total of 17,714 participants were initially recruited, and biennial follow-up was carried out. Only 7,463 individuals provided blood samples and were tested for blood lipids in 2011 and 2015, and 333 individuals did not complete the cognitive test in 2011 or 2015. We excluded 77 individuals with Alzheimer's disease, brain atrophy, or Parkinson's disease and 124 individuals who were under 45 years old at baseline. We additionally excluded 2014 participants with cognitive impairment at baseline. Eventually, 4,915 individuals with normal baseline cognition were included (characteristics between included and excluded participants are presented in Supplemental Table 1; the flowchart of participant selection is presented in Figure 1). The survey was approved by the institutional review board of Peking University, China (IRB00001052-11015). All subjects provided written informed consent at the baseline and follow up.

### Cholesterol Measurement and Assessment

A blood sample was collected after an overnight fast by medically trained staff of the local Centres for Disease Control and Prevention (CDC) at the survey site. Whole blood (4 mL) was centrifuged into plasma and buffy coat and then stored in cryovials and frozen at −20°C. Plasma and buffy coat were transported within 2 weeks to the Chinese CDC in Beijing, where they were placed in a deep freezer and stored at −80°C. Plasma cholesterol was determined by an enzymatic colorimetric test in the Youanmen Center for Clinical Laboratory of Capital Medical University. Non-high-density lipoprotein cholesterol (NHDL-C) was calculated by subtracting HDL-C from TC. The high levels of TC, NHDL-C, LDL-C, and HDL-C were defined as >240, >190, >160, and >50 mg/dL, respectively, according to the guidelines for the prevention and treatment of dyslipidaemia in Chinese adults (revised in 2016) (Opoku et al., 2019). Blood tests were only carried out in 2011 and 2015. Variation in cholesterol was classified into four groups: low–low, low levels in 2011 and 2015; low–high, low level in 2011 and high level in 2015; high–low, high level in 2011 and low level in 2015; and high–high, high levels in 2011 and 2015. Participants in the low–high and high–low groups both had higher intraindividual CV and SD, and participants in the low–low and high–high groups both had lower intraindividual CV and SD (Supplementary Figure 1).

### Cognitive Assessment

In CHARLS, cognitive function was evaluated through episodic memory and mental intactness. For episodic memory, each participant was asked to repeat as many words as possible immediately after the investigator read a list of 10 words (immediate word recall) and after 5 min (delayed word recall). One correct word recall was coded as one point. Episodic memory score was 20 points in total. The method of episodic memory measurement in this survey has good reliability and validity (Baars et al., 2009).

Numerical ability, time orientation, and picture drawing were used to assess mental intactness. For numerical ability, each participant was asked to subtract 7 from 100 serially five times. Time orientation was assessed by asking the participant to provide the date of the investigation day (month, day, and year), day of the week, and season of the year. For picture drawing, the participants were shown a picture of two overlapping pentagons and asked to redraw it. Same as episodic memory, one correct answer or successful redraw of the picture was coded as one point. The mental intactness score ranged from 0 to 11 points. Mental intactness is a well-established and valid measure as the Mini-Mental State Examination used to screen cognitively impaired individuals (Seo et al., 2011).

Global cognitive score was the summation of episodic memory and mental intactness scores and ranged from 0 to 31. Cognitive impairment was defined as a score of fewer than 11 points according to previous studies (Crimmins et al., 2011; Zhou et al., 2020). In addition, the participants with the lowest quartiles of difference in episodic memory or mental intactness score from 2011 to 2015 were considered to have a remarkable decline in memory function and mental intactness, respectively. Cognitive decline was, thus, determined by global cognitive decline, episodic memory decline, or mental intactness decline in 2015.

### Covariates

Covariates were collected by questionnaires in the baseline survey. Education level was categorized as illiterate, primary school, primary or private school, middle school, high school, or above. Marital status was grouped as married and living together, married but separated and single (divorced, widowed, or never married). Smoking and drinking were arranged as all the time, former, and never. Active exercise was defined as light intensity physical activity at least twice a week. According to Chinese body mass index (BMI) standards, underweight, normal weight, and overweight/obesity were defined as BMI <18.5, 18.5–23.9, and ≥24.0 kg/m^2^, respectively. Physical diseases were based on self-report as a response to the question, “Has a doctor ever told you that you had hypertension, diabetes, disability, heart problems, or stroke?” Medication use was also investigated by self-report. In addition, systolic pressure ≥140 mmHg or diastolic pressure ≥90 mmHg at baseline was also considered hypertension, and fasting blood glucose ≥126 mg/dL or glycosylated haemoglobin ≥6.5% was also considered as diabetes.

### Statistical Analyses

Baseline characteristics between participants with and without global cognitive decline were presented. Unordered categorical variables were compared using χ^2^-test, and continuous variables with normal distribution were compared by Student's *t*-test. The non-parametric test Kruskal–Wallis was used for comparing the differences in ordinal, categorical, and continuous variables with non-normal distribution between groups.

Binary logistic regression was used to evaluate the association of cholesterol variation with cognitive decline. We estimated the odds ratio (OR) and 95% confidence interval (CI) of the low–high and high–high groups using the low-low group as the reference; then, we also estimated the OR and 95% CI of the high–low group with the high–high group as the reference. We conducted three statistical models. The initial model was not adjusted for any covariates; the second model was adjusted for age, sex, education, marital status, smoking, drinking, exercise, BMI, diabetes, and history of disability; the last model was adjusted the same as the second model plus hypertension, heart problems (myocardial infarction, coronary heart disease, angina pectoris, heart failure, other heart diseases), stroke, and medication use (antihypertensive or antidiabetic medications). In addition, we also adjusted the number of comorbidities in the third model, which was composed of hypertension, diabetes, disability, heart problems, and stroke, because multiple morbidities are relevant to cognitive impairment.

In the sex subgroup analyses, menstrual status was adjusted in the female subgroup additionally. The subgroup analyses according to the presence or absence of cardiovascular disease was only performed in the first two statistical models. We also conducted sensitivity analyses by separating individuals with hypertension only and those with heart problems or stroke to further confirm the association between cholesterol and cognitive decline in people with different cardiovascular diseases. Null covariates were imputed by bootstrapped multiple imputation.

All statistical tests were two-sided, and the significance level was *P* < 0.05. Statistical analyses were performed using SAS software version 9.4 (SAS Institute Inc., Cary, NC).

## Results

### Characteristics of the Participants

Among the 4,915 cognitively normal participants in 2011, 979 (19.91%) had global cognitive decline in 2015. The mean ± SD of age was 57.7 ± 8.2 years, and 49.2% of the participants were females. The participants with incident global cognitive decline were older, had lower BMI, lower education level, and higher baseline HDL-C. The proportions of individuals who were female, single and with history of disability and without drinking history were higher in the group with incident global cognitive decline. Smoking status; history of cardiovascular diseases or stroke; and baseline TC, NHDL-C, and LDL-C were not remarkably different between the participants with and without incident global cognitive decline. Females with menopause were also more likely to experience global cognitive decline (Table 1).

**Table 1:** Baseline characteristics of participants with or without cognitive decline from 2011 to 2015.

**Characteristics**	**Total**	**Persistent normal**	Cognitive decline^*^	***P***
*n* (%)	4,915	3,936 (80.1)	979 (19.9)	
Age, years (mean ± SD)	57.7 ± 8.2	56.8 ± 7.8	61.3 ± 8.9	<0.001
Female, *n* (%)	2,416 (49.2)	1,874 (47.6)	542 (55.4)	<0.001
Menopause, *n* (%)^#^	1,676 (69.4)	1,231 (65.7)	445 (82.1)	<0.001
BMI, kg/m^2^ (mean ± SD)	23.9 ± 3.7	24.1 ± 3.7	23.1 ± 3.4	<0.001
BMI, *n* (%)				<0.001
Underweight	209 (4.3)	138 (3.5)	71 (7.3)	
Normal weight	2,719 (55.3)	2,139 (54.3)	580 (59.2)	
Overweight or obesity	1,987 (40.4)	1,659 (42.2)	328 (33.5)	
Marital status, *n* (%)				<0.001
Married, living together	4,324 (88.0)	3,530 (89.7)	794 (81.1)	
Married, separated	179 (3.6)	142 (3.6)	37 (3.8)	
Single	412 (8.4)	264 (6.7)	148 (15.1)	
Education, *n* (%)				<0.001
Illiterate	811 (16.5)	410 (10.4)	401 (41.0)	
Part of primary school	878 (17.9)	656 (16.7)	222 (22.7)	
Primary or private school	1,333 (27.1)	1,100 (27.9)	233 (23.8)	
Middle school	1,274 (25.9)	1,172 (29.8)	102 (10.4)	
High school or above	619 (12.6)	598 (15.2)	21 (2.2)	
Smoking, *n* (%)				0.101
Never smoking	2,879 (58.6)	2,281 (58.0)	598 (61.1)	
Former smoking	331 (6.7)	277 (7.0)	54 (5.5)	
All the time	1,705 (34.7)	1,378 (35.0)	327 (33.4)	
Drinking, *n* (%)				0.001
Never drinking	3,276 (66.7)	2,600 (66.1)	676 (69.1)	
Former drinking	308 (6.3)	231 (5.9)	77 (7.9)	
All the time	133 (27.1)	1,105 (28.1)	226 (23.1)	
Active exercise, *n* (%)	927 (18.9)	751 (19.1)	176 (18.0)	0.430
Disability, *n* (%)	668 (13.6)	475 (12.1)	193 (19.7)	<0.001
Hypertension, *n* (%)	1,849 (37.6)	1,456 (37.0)	393 (40.1)	0.069
Diabetes, *n* (%)	705 (14.3)	567 (14.4)	138 (14.1)	0.805
Heart problems, *n* (%)	583 (11.9)	478 (12.1)	105 (10.7)	0.219
Stroke, *n* (%)	84 (1.7)	65 (1.6)	19 (1.9)	0.532
Medication use, *n* (%)	540 (11.0)	418 (10.6)	122 (12.5)	0.099
TC	193.1 ± 37.2	192.8 ± 37.2	194.2 ± 37.4	0.304
NHDL-C	142.9 ± 37.5	142.9 ± 37.5	142.7 ± 37.4	0.842
LDL-C	116.1 ± 34.6	116.2 ± 34.4	115.6 ± 35.2	0.591
HDL-C	50.2 ± 15.1	49.8 ± 15.0	51.5 ± 15.3	0.002

* *Cognitive decline was determined by global cognitive function;*

# *Proportion of menopause was calculated in females. BMI, body mass index; SD, standard deviation; TC, total cholesterol; NHDL-C, non-high-density lipoprotein cholesterol; LDL-C, low-density lipoprotein cholesterol; HDL-C, high-density lipoprotein cholesterol; OR, odds ratio; CI, confidence interval*.

**Figure 2:**
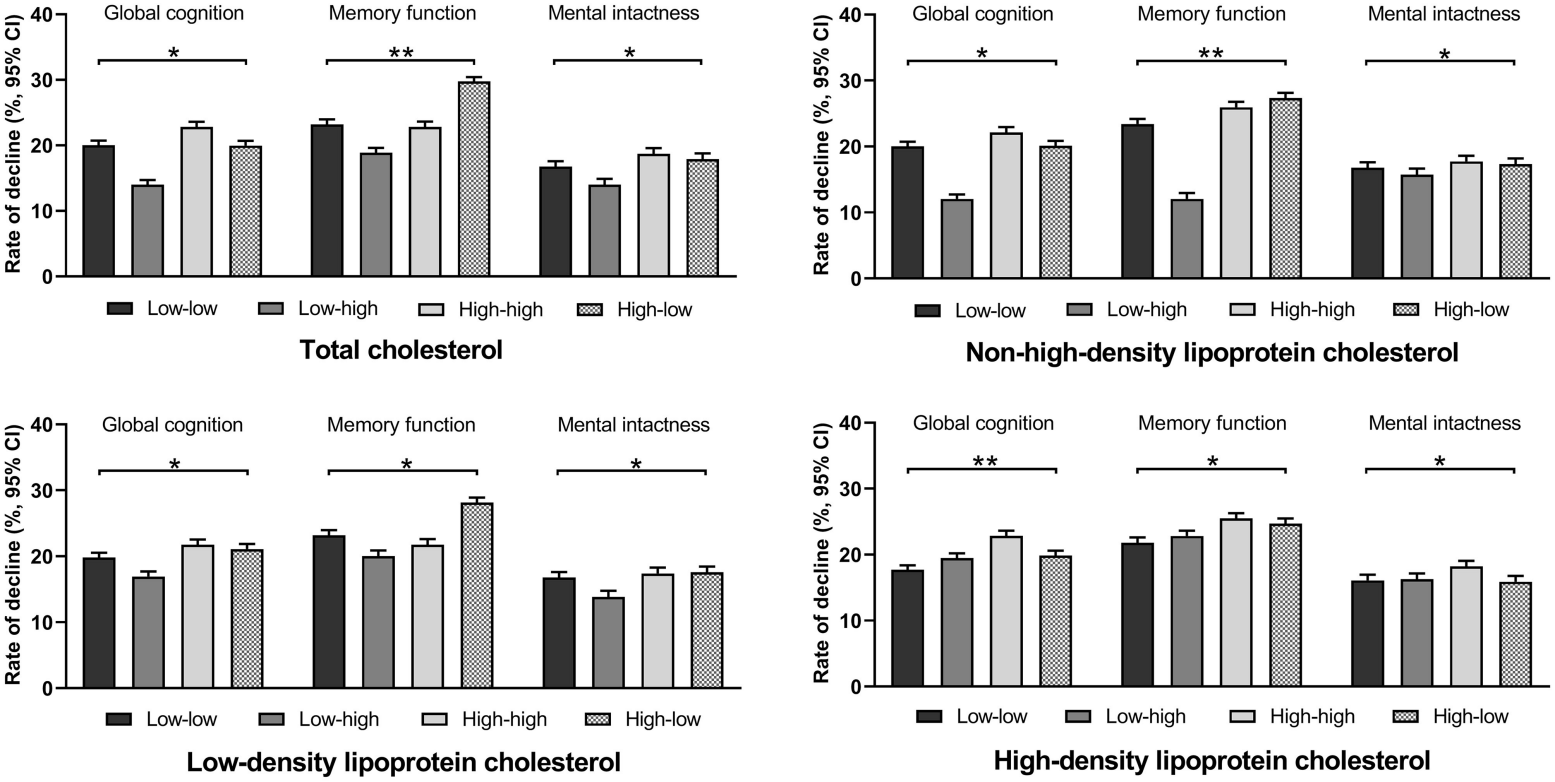
Incidence rate of cognitive decline among cholesterol groups. *P > 0.05; **P < 0.05.

The incidence of memory function decline was lower in the low–high TC or low–high NHDL-C group, and the incidence of global cognitive decline was higher in the high–high HDL-C group. No remarkable difference in the incidence of cognitive decline was observed in the other variation groups of cholesterol (Figure 2).

Females were more likely to have abnormal cholesterol with larger proportions in the low–high and high–high cholesterol groups than males (*P* < 0.05). TC and NHDL-C levels were more likely to decrease in participants with at least one cardiovascular disease, and LDL-C and HDL-C were more likely to decrease in participants without any cardiovascular disease (Supplemental Table 2).

### Cholesterol Variation and Cognitive Decline in All Participants

**Figure 3:**
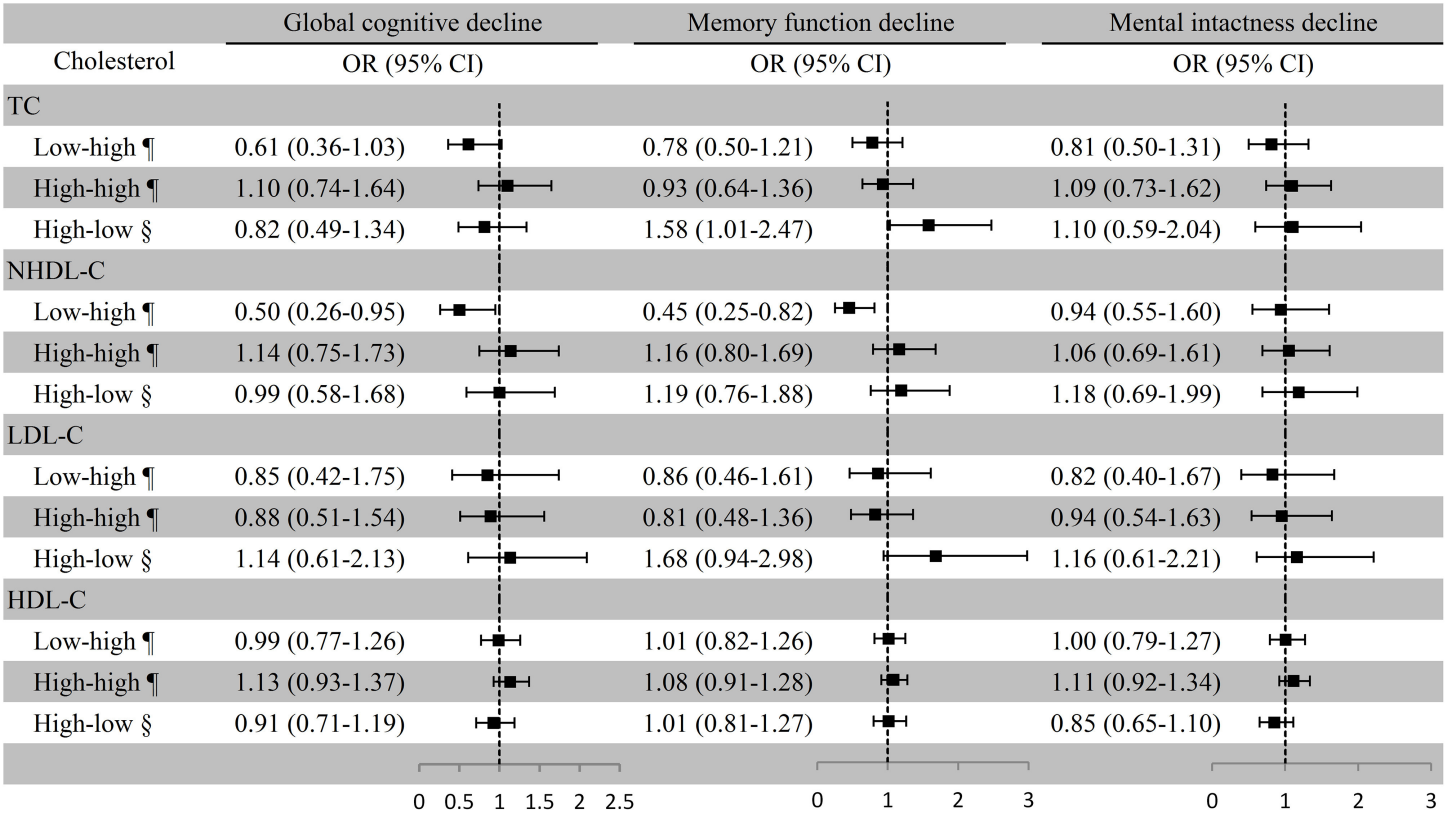
Association between cholesterol variation and cognitive decline among all the participants. ^¶^Take the low–low group as a reference; ^§^Take the high–high group as a reference. Baseline age, sex, education, marital status, smoking, drinking, BMI, exercise, physical diseases, medication use (antihypertensive or antidiabetic medications), and the number of comorbidities were adjusted. TC, total cholesterol; NHDL-C, non-high-density lipoprotein cholesterol; LDL-C, low-density lipoprotein cholesterol; HDL-C, high-density lipoprotein cholesterol; OR, odds ratio; CI, confidence interval.

After multivariate adjustment, the risk of global cognitive decline for the low–high TC group was borderline significant (OR [95% CI]: 0.61 [0.36–1.03]) compared with the low–low TC group. The risk of memory function decline for the high–low TC group was significantly increased (OR [95% CI]: 1.58 [1.01–2.47]) compared with the high–high TC group. The OR and 95% CI of global cognitive decline and memory function decline for the low–high NHDL-C group were 0.50 (0.26–0.95) and 0.45 (0.25–0.82), respectively, with the low–low NHDL-C group as the reference. Cognitive decline was not remarkably associated with LDL-C and HDL-C variation in all participants after adjusted covariates (Figure 3). In the first and second statistical models, the association of LDL-C and NHDL-C with cognitive decline was in line with that in the third model. However, the TC variation was not significantly associated with cognitive decline. The persistent high level of HDL-C was positively and significantly associated with global cognitive decline and memory function decline in the first model (Supplemental Table 3).

### Cholesterol Variation and Cognitive Decline in the Sex Subgroups

**Figure 4:**
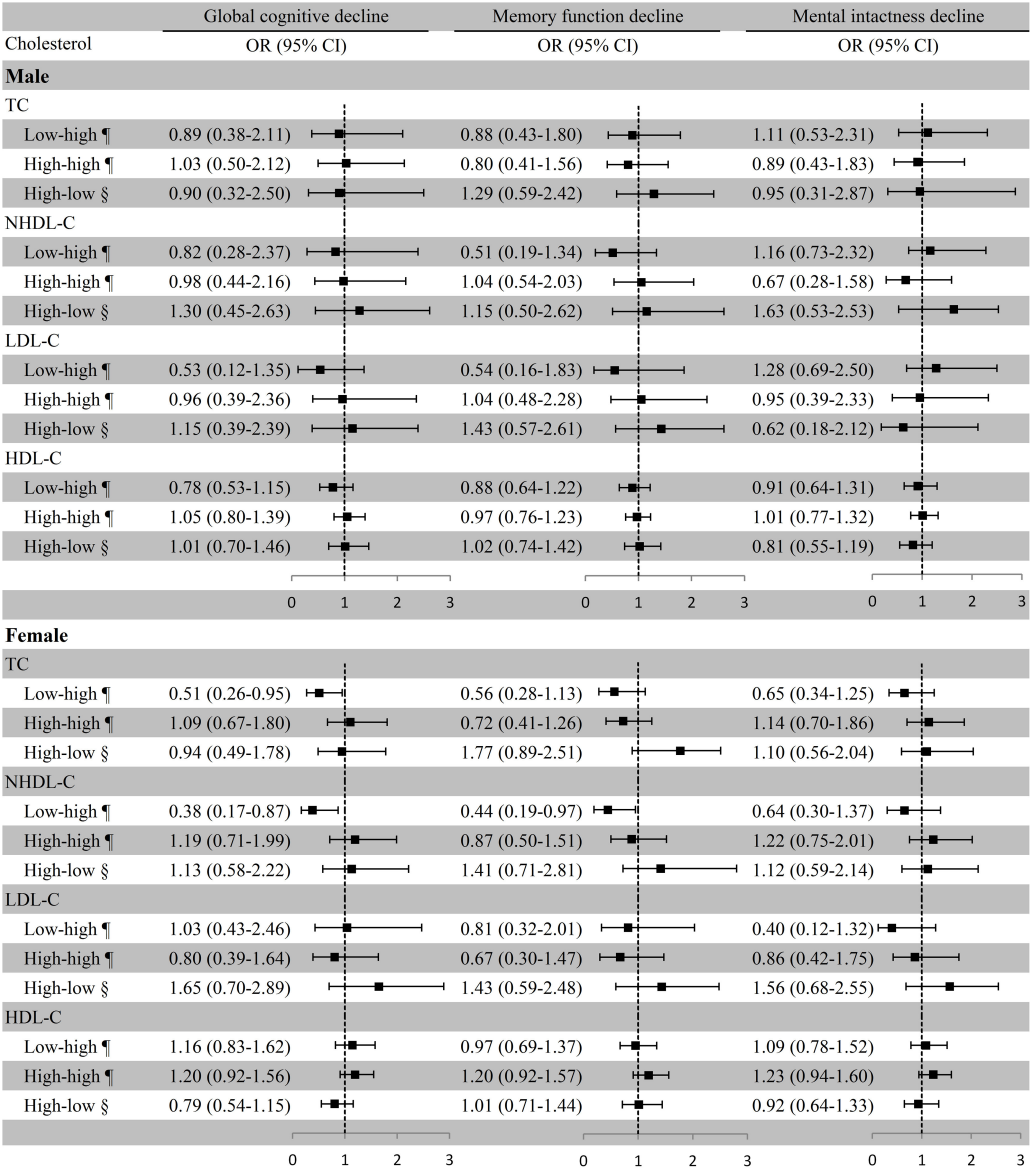
Association between cholesterol variation and cognitive decline in sex subgroups. ^¶^Take the low–low group as a reference; ^§^Take the high–high group as a reference. Baseline age, education, marital status, smoking, drinking, BMI, exercise, physical diseases, medication use (antihypertensive or antidiabetic medications), and the number of comorbidities were adjusted. Menstrual status was also adjusted in the female subgroup. TC, total cholesterol; NHDL-C, non-high-density lipoprotein cholesterol; LDL-C, low-density lipoprotein cholesterol; HDL-C, high-density lipoprotein cholesterol; OR, odds ratio; CI, confidence interval.

In females, substantially lower risks of global cognitive decline (OR [95% CI]: 0.38 [0.17–0.87]) and memory function decline (OR [95% CI]: 0.44 [0.19–0.97]) were observed in the low–high NHDL-C group compared with the low–low NHDL-C group, which was used as the reference in the multivariate adjustment model; remarkably lower risk of global cognitive decline was also observed in the low–high TC group with the low–low group as the reference (OR [95% CI]: 0.51 [0.26–0.95]). However, the associations of cholesterol variation with cognitive decline were not substantial in males (Figure 4). In the first model of the female subgroup, the increase in TC was not associated with global cognitive decline, and the persistent high level of HDL-C was positively associated with global cognitive decline and memory function decline although not significant in the third model. The association of LDL-C and NHDL-C with cognitive decline was in line with that in the third model. Consistent conclusions were found among the three models of the male subgroup (Supplemental Table 4).

### Cholesterol Variation and Cognitive Decline in Participants With or Without Cardiovascular Disease

**Figure 5:**
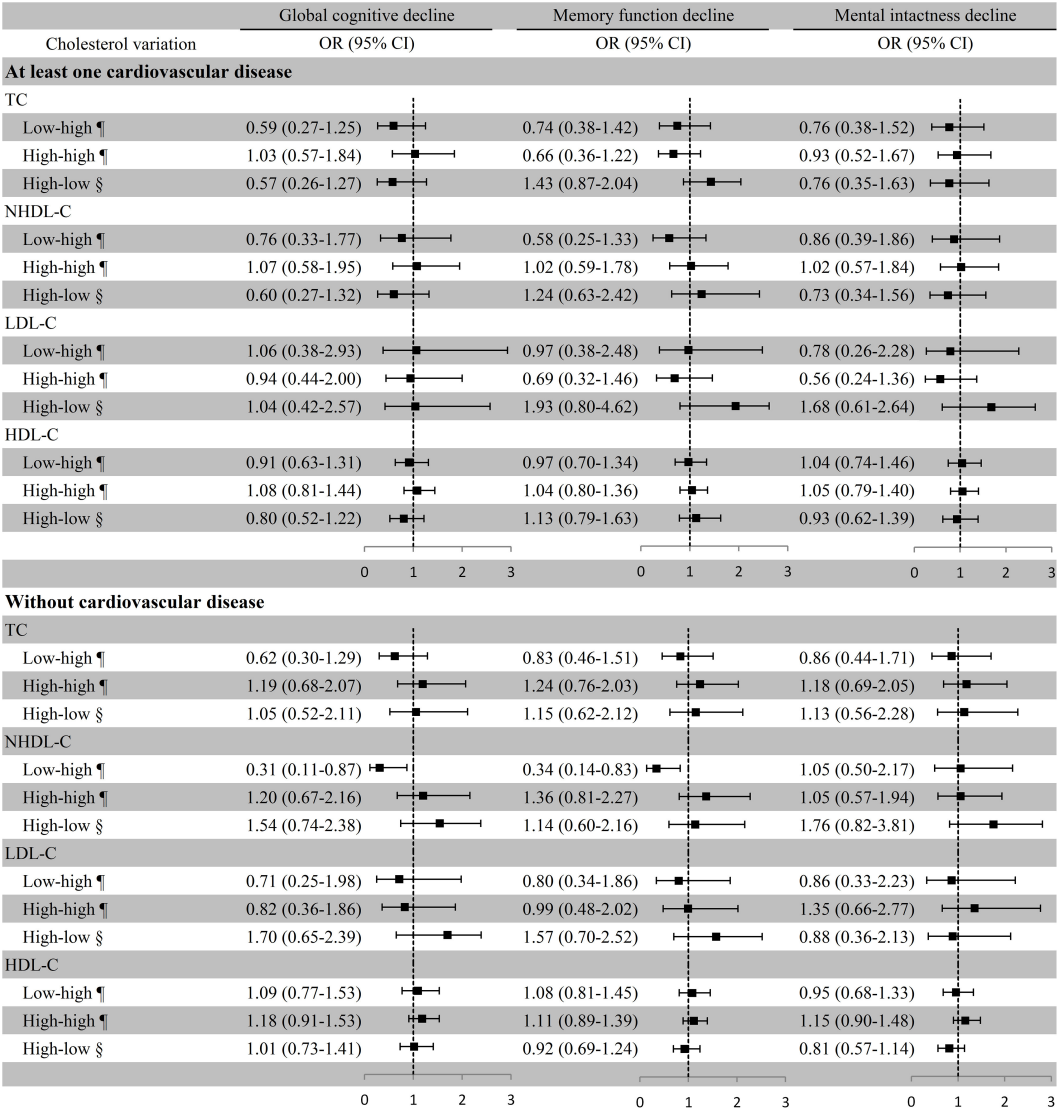
Association between cholesterol variation and cognitive decline in participants with or without cardiovascular disease. ^¶^Take the low–low group as a reference; ^§^Take the high–high group as a reference. Baseline age, education, marital status, smoking, drinking, BMI, exercise, diabetes, history of disability, medication use (antihypertensive or antidiabetic medications), and the number of comorbidities were adjusted. TC, total cholesterol; NHDL-C, non-high-density lipoprotein cholesterol; LDL-C, low-density lipoprotein cholesterol; HDL-C, high-density lipoprotein cholesterol; OR, odds ratio; CI, confidence interval.

In participants without cardiovascular disease, remarkably lower risk of global cognitive decline (OR [95% CI]: 0.31 [0.11–0.87]) and memory function decline (OR [95% CI]: 0.34 [0.14–0.83]) were observed in the low–high NHDL-C group with the low–low group as the reference in the multivariate adjustment model, but global cognitive decline was not remarkably associated with TC variation. The associations of cholesterol variation with any cognitive decline were not substantial in participants with at least one cardiovascular disease (Figure 5). Similar results were also observed in the first statistical model for LDL-C and NHDL-C and cognitive decline. In addition, we observed a considerable increase in the ORs of global cognitive decline (OR [95% CI]: 1.51 [1.23–1.84]) and memory function decline (OR [95% CI]: 1.24 [1.03–1.49]) in the high–high HDL-C group with the low–low group as a reference in participants without any cardiovascular disease although it was not significant in the adjusted model (Supplemental Table 5).

We further stratified participants into hypertension only and with heart problems or stroke. The results in both groups were similar to those in participants with at least one cardiovascular disease (Supplemental Table 6).

## Discussion

We estimated the risk of cognitive decline based on the long-term variation patterns and relatively stable levels of cholesterol. The results show that the long-term increase in TC and NHDL-C levels were substantially associated with decreased risks of global cognitive and memory function decline. Substantial associations were mainly present in females and participants without any cardiovascular disease. The long-term decrease in cholesterol level had no protective effect on cognition. Persistent high cholesterol levels did not remarkably increase the risk of any cognitive dimensions compared with persistent low levels.

Cholesterol is a well-established risk factor for cardiovascular diseases (Brunner et al., 2019). Cardiovascular health is helpful in keeping normal cognition, and the role of cholesterol on cognition should be in line with cardiovascular disease. However, the association between cholesterol and cognitive function has been divergent in recent years. A study on a Chinese urban community indicated that higher LDL-C is associated with lower dementia risk (Zhou et al., 2018). An ideal TC level, one of seven metrics of ideal vascular health, was negatively associated with cognition in the Brazilian Longitudinal Study of Adult Health (Suemoto et al., 2021). A randomized trial in hypercholesterolemic adults also indicates that cholesterol-lowering therapy by simvastatin diminishes cognitive function slightly compared with placebo (Muldoon et al., 2004). In addition, in animal experiments, the cholesterol infusion to striatum of Huntington's disease mice prevented cognitive decline (Birolini et al., 2020). Two pieces of evidence can explain the divergent findings. First, approximately a quarter of the body's cholesterol is concentrated in the brain, and lipid composition, including lecithin, omega-3, and cholesterol, account for nearly half of the brain's weight. Although peripheral cholesterol cannot enter the central nervous system because of the blood–brain barrier, it reflects the supplement of cholesterol (Dai et al., 2021). Crucially, cholesterol is an important component of nerve cell membranes and also participates in the metabolic activities of nerve cells. Another factor is that cholesterol stores a large amount of energy, which can be sustainably provided to the brain for a long time, and the brain is the most energy-consuming organ of the body (Steiner, 2019). Therefore, the role of cholesterol in brain protection might be different from its role in cardiovascular diseases.

Previous studies clarify the associations of higher visit-to-visit cholesterol with cognitive decline (Smit et al., 2016; Grasset et al., 2020; Hua et al., 2020). In these studies, CV, VIM, and SD reflect the degree of intraindividual dispersion of cholesterol, including downward and upward fluctuations. One possible phenomenon is that the intraindividual CV, VIM, or SD of cholesterol in recommended and high ranges are comparable and, thus, collapse persistent high and low cholesterol into the same category. In our cholesterol groups, intraindividual SD and CV were higher in low–high and high–low groups and lower in low–low and high–high groups. Thus, we estimate the risk of cognitive decline from another perspective, including persistently low levels, levels that cross the diagnostic threshold, and persistently high levels. We found that variations in TC and NHDL-C levels from low to high have a remarkable protective role on cognitive function, whereas changes from high to low levels do not have the same effect.

Hua et al. (2020) find a negative association between the intraindividual SD of TC and memory function in males using the same database as our present study. Stable cholesterol is certainly favourable for cognitive ability. However, we did not observe a remarkable association between cholesterol variation and any cognitive domains in our male subgroup analyses. Hua et al. suggest the importance of maintaining a stable cholesterol level, whereas our study adds certain guiding importance on whether to use lipid-lowering intervention. This difference indicates that different grouping methods have different guiding importance for the prevention of cognitive decline.

Some studies clarify the benefit of increased TC on cognitive function. A study based on the Finland population indicates that a moderate decrease in serum TC from mid- to late life is associated with a 3.5-fold increase in the risk of cognitive impairment (Solomon et al., 2007). The same conclusion was documented in Swedish females, in which the greatest decrease in TC (refers to the lowest quartile of TC difference) after 32 years of follow-up is associated with a 2.37-fold increase in the risk of dementia (Mielke et al., 2010). In a birth cohort study from Germany, TC level from baseline to endpoint decreased in participants with Alzheimer's disease but remained stable in those who remained healthy (Toro et al., 2014). These studies suggest that TC is protective for cognitive function, and this conclusion is consistent with our study. However, the assessment of the variation trend of cholesterol was not based on baseline cholesterol level in these studies; thus, participants with low and high baseline cholesterol levels might have the same decrease. We conducted two reference groups, the low–low and high–high groups, to address this issue. Furthermore, we demonstrated that the results for NHDL-C are still the same after we subtracted HDL-C from TC. LDL-C is the main component of NHDL-C. However, the variation in LDL-C was not associated with cognitive function in our study. Therefore, in addition to LDL-C, the effect of other lipid compositions on cognition needs to be further determined.

Menstruation in females is of importance for cognitive decline. The decrease of estrogen levels in postmenopausal females results in low cholesterol ester transfer protein and disturbs the reverse transport of cholesterol to the liver and the metabolism of cholesterol (Guo et al., 2019). Previous studies report an increased risk of decline in psychomotor speed in females who never used hormone treatment compared with current users (Ryan et al., 2009). In our study, there were greater proportions of females in the low–high and high–high cholesterol groups, and the majority of these females (69.4%) were menopausal. We also adjusted menstrual status in the female subgroup. We still found a low risk of cognitive decline for increased TC or NHDL-C in females. Our results suggest that cholesterol may be an excellent protector of cognitive function even in postmenopausal females. The gender-specific association was documented elsewhere. Protease proprotein convertase subtilisin/kexin type 9 (PCSK9), a risk factor of cardiovascular diseases, is negatively associated with memory function in elderly females but not in males (Simeone et al., 2021). One reason that cannot be ignored is that estrogen is inversely correlated to PCSK9. However, we did not examine the association of cholesterol variation with cognitive decline in postmenopausal and premenopausal females because of the small number of premenopausal females.

Interestingly, the remarkable decrease in the risk of cognitive decline as NHDL-C levels change from low to high was only observed in participants without cardiovascular disease. Cardiovascular disease is a recognized risk factor for cognitive decline, and cholesterol promotes the progression of cardiovascular disease (Zlokovic et al., 2020). We speculated that cholesterol can play a better role in cognitive protection without the threat of cardiovascular disease. Our Supplementary Materials show that the levels of TC and NHDL-C are more likely to decrease in participants with at least one cardiovascular disease. Treatment for cardiovascular disease may include lipid-lowering intervention, which decreases cholesterol. This finding suggests that the pros and cons of lipid-lowering intervention should be fully weighed between cardiovascular disease and cognitive decline. The protective role of cholesterol on cognition may be covered by cardiovascular risk.

In the present study, we precisely clarify the association between cholesterol variation and cognitive decline. However, this study has several limitations. First, the blood test was only carried out in 2011 and 2015; the time of cholesterol changes is uncertain. We cannot determine the sequence of cholesterol and cognitive changes. Second, we excluded most of the participants who did not provide a blood sample in 2011 or 2015 and those who had cognitive impairment at baseline. This omission may induce selection bias. Supplemental Table 1 shows that most of the characteristics between the excluded and included participants are remarkably different. Third, we define the lowest quartile of the difference in episodic memory or mental intactness scores from 2011 to 2015 as substantial decline, which may lead to misjudgement. Memory and mental health decline have no existing standard. Fourth, information on the use of lipid-lowering drugs was not obtained in this study. However, lipid-lowering drugs, particularly statins, have controversial effects on cognition. The combined effect of cholesterol and lipid-lowering drugs on cognition needs to be further studied. Last, the existing conditions that were not officially diagnosed also resulted in information bias. In the community, not everyone could see a doctor in the time when they were sick, leading to patients not knowing that they were sick and, thus, being regarded as healthy people in the survey.

## Conclusions

In conclusion, the longitudinal increase in TC or NHDL-C is associated with better cognitive function among females or participants without cardiovascular disease. This study adds further evidence that lipid regulation strategies based on gender and cardiovascular disease diagnosis should be considered in the primary prevention of cognitive impairment.

## Data Availability Statement

Publicly available datasets were analysed in this study. This data can be found at: http://charls.pku.edu.cn/en.

## Ethics Statement

The survey was approved by the Institutional Review Board of Peking University, China (IRB00001052-11015). The patients/participants provided their written informed consent to participate in this study.

## Author Contributions

HL and LZ wrote the article. HL and RZ performed the data analysis. XW and JZ drafted and critically revised the manuscript. SG provided clinical guidance. MZ and DH reviewed language and made substantial interpretation. KW, ZH, and ZY organized database. XW contributed to the study concept and design and reviewed the article. All authors contributed to the article and approved the submitted version.

## Conflict of Interest

The authors declare that the research was conducted in the absence of any commercial or financial relationships that could be construed as a potential conflict of interest.

## Supplementary Material

Supplementary Figure 1 Intraindividual SD and CV in cholesterol variation group. SD, standard deviation; CV, coefficient of variation.

Supplemental Table 1 Characteristics between included and excluded participants.

Supplemental Table 2 Variation profile of cholesterol in subgroups.

Supplemental Table 3 The OR (95% CI) between cholesterol variation and cognitive decline amongst all the participants.

Supplemental Table 4 The OR (95% CI) between cholesterol variation and cognitive decline in sex subgroups.

Supplemental Table 5 The OR (95% CI) between cholesterol variation and cognitive decline in participants with or without cardiovascular disease.

Supplemental Table 6 The OR (95% CI) between cholesterol variation and cognitive decline in participants just with hypertension and with heart problems or stroke.
