# Supplementary figures and images for: Long-Term Increase in Cholesterol Is Associated With Better Cognitive Function: Evidence From a Longitudinal Study
Source: Front Aging Neurosci. 2021 Jun 17;13:691423. doi: 10.3389/fnagi.2021.691423 (PMC8248815; doi:10.3389/fnagi.2021.691423)

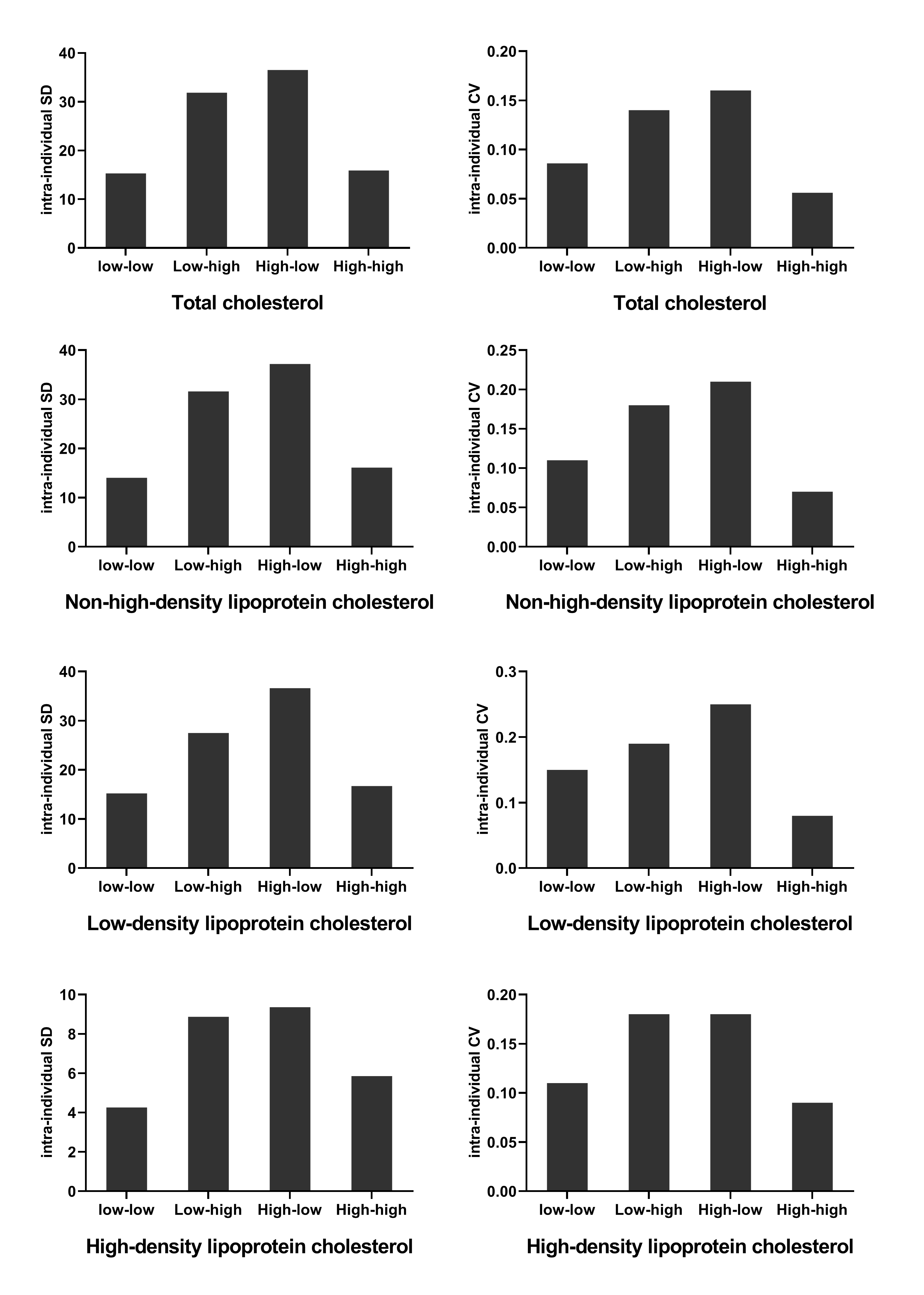

Supplement: Supplementary Figure 1 — Intraindividual SD and CV in cholesterol variation group. SD, standard deviation; CV, coefficient of variation. [file Image_1.TIF]
