# Supplementary material for: Long-Term Increase in Cholesterol Is Associated With Better Cognitive Function: Evidence From a Longitudinal Study
Source: Front Aging Neurosci. 2021 Jun 17;13:691423. doi: 10.3389/fnagi.2021.691423 (PMC8248815; doi:10.3389/fnagi.2021.691423)
Supplement: Supplemental Table 1 — Characteristics between included and excluded participants. [file Data_Sheet_1.docx]

**Long-term increase in cholesterol is associated with better cognitive function: Evidence from a** **longitudinal study**

**Supplemental Table 1** Characteristics between included and excluded participants

| Characteristics | Total | Excluded | Included | *P* |
| --- | --- | --- | --- | --- |
| n (%) | 17714 | 12799 (72.3) | 4915 (27.7) |  |
| Age, years ( mean ± SD) | 59.1 ± 10.2 | 59.6 ± 10.8 | 57.7 ± 8.2 | <0.001 |
| Female, n (%) | 9243 (52.2) | 6827 (53.3) | 2416 (49.2) | <0.001 |
| Menopause, n (%) ^‡^ | 6091 (65.9) | 4415 (64.7) | 1676 (69.4) | <0.001 |
| BMI, kg/m2 ( mean ± SD) | 23.5 ± 3.5 | 23.4 ± 3.4 | 23.9 ± 3.6 | 0.213 |
| BMI, n (%) |  |  |  | <0.001 |
| Underweight | 954 (5.4) | 745 (5.8) | 209 (4.3) |  |
| Normal weight | 11191 (63.2) | 8472 (66.2) | 2719 (55.3) |  |
| Overweight or Obesity | 5569 (31.4) | 3582 (28.0) | 1987 (40.4) |  |
| Marital status, n (%) |  |  |  | <0.001 |
| Married, living together | 14170 (80.0) | 9846 (76.9) | 4324 (88.0) |  |
| Married, separated | 1330 (7.5) | 1151 (9.0) | 179 (3.6) |  |
| Single | 2214 (12.5) | 1802 (14.1) | 412 (8.4) |  |
| Education, n (%) |  |  |  | <0.001 |
| Illiterate | 4803 (27.1) | 3992 (31.2) | 811 (16.5) |  |
| Part of primary school | 3117 (17.6) | 2239 (17.5) | 878 (17.9) |  |
| Primary or private school | 3896 (22.0) | 2563 (20.0) | 1333 (27.1) |  |
| Middle school | 3652 (20.6) | 2378 (18.6) | 1274 (25.9) |  |
| High school or above | 2246 (12.7) | 1627 (12.7) | 619 (12.6) |  |
| Smoking, n (%) |  |  |  | <0.001 |
| Never smoking | 10682 (60.0) | 7749 (60.5) | 2879 (58.6) |  |
| Former smoking | 1892 (10.7) | 1561 (12.2) | 331 (6.7) |  |
| All the time | 5194 (29.3) | 3489 (27.3) | 1705 (34.7) |  |
| Drinking, n (%) |  |  |  | <0.001 |
| Never drinking | 12288 (69.4) | 9012 (70.4) | 3276 (66.7) |  |
| Former drinking | 1043 (5.9) | 735 (5.7) | 308 (6.3) |  |
| All the time | 4383 (24.7) | 3052 (23.9) | 133 (27.1) |  |
| Disability, n (%) | 3144 (17.6) | 2476 (19.4) | 668 (13.6) | <0.001 |
| Hypertension, n (%) | 6576 (37.1) | 4727 (36.9) | 1849 (37.6) | 0.397 |
| Diabetes, n (%) | 2070 (11.7) | 1365 (10.7) | 705 (14.3) | <0.001 |
| Heart problems, n (%) | 2093 (11.8) | 1510 (11.8) | 583 (11.9) | 0.906 |
| Stroke, n (%) | 413 (2.3) | 329 (2.6) | 84 (1.7) | <0.001 |

^‡^ Proportion of menopause was calculated in females. BMI, body mass index; SD, standard deviation.

**Supplemental Table 2** Variation profile of cholesterol in subgroups

|  | All | Male, n (%) | Female, n (%) | χ2 | *P* | At least one cardiovascular disease, n (%) | Without any cardiovascular disease, n (%) | χ2 | *P* |
| --- | --- | --- | --- | --- | --- | --- | --- | --- | --- |
| TC variation |  |  |  | 48.548 | <0.001 |  |  | 18.729 | <0.001 |
| Low-low | 4255 (86.57) | 2245 (89.84) | 2010 (83.20) |  |  | 1784 (84.72) | 2471 (88.31) |  |  |
| Low-high | 143 (2.91) | 52 (2.08) | 91 (3.77) |  |  | 69 (3.26) | 74 (2.64) |  |  |
| High-high | 171 (3.48) | 60 (2.40) | 111 (4.59) |  |  | 81 (3.83) | 90 (3.22) |  |  |
| High-low | 346 (7.04) | 142 (5.68) | 204 (8.44) |  |  | 183 (8.64) | 163 (5.83) |  |  |
| NHDL-C variation |  |  |  | 31.163 | <0.001 |  |  | 32.605 | <0.001 |
| Low-low | 4291 (87.30) | 2235 (89.44) | 2056 (85.10) |  |  | 1787 (84.41) | 2504 (89.49) |  |  |
| Low-high | 108 (2.20) | 40 (1.60) | 68 (2.81) |  |  | 50 (2.36) | 58 (2.07) |  |  |
| High-high | 158 (3.21) | 53 (2.12) | 105 (4.35) |  |  | 78 (3.68) | 80 (2.86) |  |  |
| High-low | 358 (7.28) | 171 (6.84) | 187 (7.74) |  |  | 202 (9.54) | 156 (5.58) |  |  |
| LDL-C variation |  |  |  | 33.663 | <0.001 |  |  | 15.617 | 0.001 |
| Low-low | 4360 (88.71) | 2281 (91.28) | 2079 (86.05) |  |  | 1837 (86.77) | 2523 (90.17) |  |  |
| Low-high | 65 (1.32) | 24 (0.96) | 41 (1.70) |  |  | 28 (1.32) | 37 (1.32) |  |  |
| High-high | 92 (1.87) | 37 (1.48) | 55 (2.28) |  |  | 46(2.17) | 46 (1.64) |  |  |
| High-low | 398 (8.10) | 157 (6.28) | 241 (9.98) |  |  | 206 (1.64) | 192 (6.86) |  |  |
| HDL-C variation |  |  |  | 58.858 | <0.001 |  |  | 53.686 | <0.001 |
| Low-low | 2013 (40.96) | 1147 (45.90) | 866 (35.84) |  |  | 973 (45.96) | 1040 (37.17) |  |  |
| Low-high | 688 (14.00) | 308 (12.32) | 380 (15.73) |  |  | 314 (14.83) | 374 (13.37) |  |  |
| High-high | 1635 (33.27) | 745 (29.81) | 890 (36.84) |  |  | 603 (28.48) | 1032 (36.88) |  |  |
| High-low | 579 (11.78) | 299 (11.96) | 280 (11.59) |  |  | 227 (10.72) | 352 (12.58) |  |  |

TC, total cholesterol; NHDL-C, non-high-density lipoprotein cholesterol; LDL-C, low-density lipoprotein cholesterol; HDL-C, high-density lipoprotein cholesterol.

**Supplemental Table 3** The OR (95% CI) between cholesterol variation and cognitive decline amongst all the participants

| Cholesterol variation | Global cognitive decline | |  | Memory function decline | |  | Mental intactness decline | |
| --- | --- | --- | --- | --- | --- | --- | --- | --- |
|  | Model 1 | Model 2 |  | Model 1 | Model 2 |  | Model 1 | Model 2 |
| TC |  |  |  |  |  |  |  |  |
| Low-high ¶ | 0.65 (0.40-1.05) | 0.61 (0.34-1.03) |  | 0.77 (0.50-1.18) | 0.76 (0.46-1.15) |  | 0.81 (0.50-1.31) | 0.81 (0.51-1.34) |
| High-high ¶ | 1.18 (0.82-1.70) | 1.11 (0.76-1.64) |  | 0.98 (0.68-1.41) | 0.93 (0.65-1.36) |  | 1.15 (0.77-1.69) | 1.10 (0.74-1.64) |
| High-low § | 0.84 (0.54-1.31) | 0.84 (0.52-1.38) |  | 1.43 (0.94-2.19) | 1.52 (0.97-2.36) |  | 0.95 (0.59-1.52) | 0.99 (0.54-1.63) |
| NHDL-C |  |  |  |  |  |  |  |  |
| Low-high ¶ | 0.51 (0.27-0.96) | 0.56 (0.33-0.99) |  | 0.45 (0.25-0.80) | 0.45 (0.23-0.80) |  | 0.93 (0.55-1.57) | 0.95 (0.53-1.61) |
| High-high ¶ | 1.14 (0.75-1.74) | 1.13 (0.76-1.66) |  | 1.15 (0.80-1.65) | 1.16 (0.80-1.68) |  | 1.07 (0.71-1.62) | 1.03 (0.67-1.60) |
| High-low § | 0.96 (0.57-1.60) | 0.85 (0.52-1.41) |  | 1.08 (0.70-1.65) | 1.13 (0.72-1.75) |  | 0.97 (0.60-1.59) | 1.06 (0.63-1.77) |
| LDL-C |  |  |  |  |  |  |  |  |
| Low-high ¶ | 0.82 (0.43-1.58) | 0.84 (0.40-1.72) |  | 0.83 (0.45-1.53) | 0.84 (0.43-1.60) |  | 0.80 (0.39-1.62) | 0.81 (0.40-1.67) |
| High-high ¶ | 1.12 (0.68-1.85) | 0.93 (0.51-1.57) |  | 0.92 (0.56-1.52) | 0.83 (0.49-1.37) |  | 1.05 (0.61-1.80) | 0.94 (0.55-1.68) |
| High-low § | 0.96 (0.56-1.67) | 1.11 (0.60-2.05) |  | 1.41 (0.82-2.42) | 1.66 (0.92-2.90) |  | 1.01 (0.56-1.84) | 1.07 (0.56-2.01) |
| HDL-C |  |  |  |  |  |  |  |  |
| Low-high ¶ | 1.13 (0.93-1.40) | 0.98 (0.77-1.26) |  | 1.06 (0.86-1.30) | 1.01 (0.82-1.26) |  | 1.01 (0.80-1.28) | 0.98 (0.77-1.26) |
| High-high ¶ | 1.38 (1.17-1.62) | 1.10 (0.90-1.37) |  | 1.23 (1.05-1.43) | 1.05 (0.92-1.25) |  | 1.16 (0.98-1.38) | 1.11 (0.93-1.33) |
| High-low § | 0.84 (0.66-1.06) | 0.88 (0.69-1.14) |  | 0.96 (0.77-1.19) | 0.89 (0.73-1.24) |  | 0.85 (0.66-1.10) | 0.84 (0.65-1.11) |

Model 1: unadjusted;

Model 2: adjusted for baseline age, education, marital status, smoking, drinking, BMI and exercise.

¶ Take the low-low group as a reference; § Take the high-high group as a reference.

TC, total cholesterol; NHDL-C, non-high-density lipoprotein cholesterol; LDL-C, low-density lipoprotein cholesterol; HDL-C, high-density lipoprotein cholesterol.

**Supplemental Table 4** The OR (95% CI) between cholesterol variation and cognitive decline in sex subgroups

| Cholesterol variation | Global cognitive decline | |  | Memory function decline | |  | Mental intactness decline | |
| --- | --- | --- | --- | --- | --- | --- | --- | --- |
|  | Model 1 | Model 2 |  | Model 1 | Model 2 |  | Model 1 | Model 2 |
| **Male** |  |  |  |  |  |  |  |  |
| TC |  |  |  |  |  |  |  |  |
| Low-high ¶ | 0.72 (0.32-1.61) | 0.90 (0.39-2.11) |  | 0.81 (0.40-1.62) | 0.86 (0.44-1.75) |  | 1.07 (0.52-2.22) | 1.10 (0.53-2.31) |
| High-high ¶ | 0.93 (0.46-1.84) | 1.03 (0.50-2.15) |  | 0.76 (0.39-1.48) | 0.74 (0.41-1.55) |  | 0.90 (0.44-1.85) | 0.95 (0.45-1.88) |
| High-low § | 0.87 (0.38-1.97) | 0.98 (0.37-2.68) |  | 1.17 (0.89-2.04) | 1.16 (0.98-2.07) |  | 0.82 (0.35-1.95) | 0.91 (0.32-2.53) |
| NHDL-C |  |  |  |  |  |  |  |  |
| Low-high ¶ | 0.66 (0.26-1.69) | 0.84 (0.28-2.43) |  | 0.48 (0.19-1.22) | 0.51 (0.19-1.35) |  | 1.49 (0.70-3.15) | 1.56 (0.71-2.93) |
| High-high ¶ | 0.82 (0.38-1.75) | 1.19 (0.45-3.13) |  | 0.98 (0.51-1.87) | 1.05 (0.54-2.05) |  | 0.66 (0.27-1.54) | 0.71 (0.33-1.52) |
| High-low § | 1.01 (0.43-2.38) | 0.99 (0.46-2.18) |  | 1.08 (0.52-2.24) | 1.05 (0.47-2.32) |  | 1.22 (0.47-3.17) | 1.34 (0.75-3.98) |
| LDL-C |  |  |  |  |  |  |  |  |
| Low-high ¶ | 0.43 (0.10-1.82) | 0.55 (0.14-2.32) |  | 0.49 (0.14-1.64) | 0.55 (0.17-1.87) |  | 1.12 (0.68-2.05) | 1.12 (0.72-2.01) |
| High-high ¶ | 1.09 (0.48-1.51) | 0.98 (0.40-2.40) |  | 1.09 (0.51-2.33) | 1.05 (0.47-2.23) |  | 1.00 (0.41-2.41) | 0.94(0.39-2.29) |
| High-low § | 0.89 (0.35-2.24) | 1.07 (0.40-2.90) |  | 1.21 (0.53-2.77) | 1.60 (0.65-2.93) |  | 0.75 (0.28-2.03) | 0.69 (0.22-2.04) |
| HDL-C |  |  |  |  |  |  |  |  |
| Low-high ¶ | 0.84 (0.58-1.20) | 0.77 (0.52-1.13) |  | 0.93 (0.68-1.26) | 0.86 (0.60-1.20) |  | 0.94 (0.66-1.32) | 0.91 (0.64-1.30) |
| High-high ¶ | 1.38 (1.09-1.75) | 1.03 (0.78-1.36) |  | 1.16 (0.93-1.44) | 0.98 (0.76-1.23) |  | 1.08 (0.84-1.38) | 1.00 (0.76-1.33) |
| High-low § | 0.94 (0.67-1.32) | 0.99 (0.69-1.42) |  | 0.99 (0.72-1.34) | 0.99 (0.72-1.37) |  | 0.81 (0.6-1.18) | 0.79 (0.54-1.18) |
| **Female** |  |  |  |  |  |  |  |  |
| TC |  |  |  |  |  |  |  |  |
| Low-high ¶ | 0.57 (0.32-1.04) | 0.50 (0.26-0.98) |  | 0.58 (0.30-1.13) | 0.56 (0.28-1.12) |  | 0.66 (0.35-1.26) | 0.64 (0.34-1.23) |
| High-high ¶ | 1.22 (0.79-1.89) | 0.95 (0.51-1.81) |  | 0.79 (0.46-1.36) | 0.71 (0.41-1.26) |  | 1.26 (0.78-2.02) | 1.11 (0.70-1.87) |
| High-low § | 0.87 (0.51-1.48) | 1.12 (0.69-1.82) |  | 1.58 (0.85-2.96) | 1.70 (0.81-2.30) |  | 1.05 (0.60-1.86) | 1.12 (0.62-2.07) |
| NHDL-C |  |  |  |  |  |  |  |  |
| Low-high ¶ | 0.46 (0.22-0.97) | 0.39 (0.18-0.84) |  | 0.46 (0.20-1.00) | 0.43 (0.17-0.97) |  | 0.64 (0.30-1.35) | 0.64 (0.30-1.38) |
| High-high ¶ | 1.20 (0.76-1.88) | 1.17 (0.70-1.96) |  | 0.91 (0.54-1.55) | 0.87 (0.51-1.50) |  | 1.27 (0.79-2.07) | 1.20 (0.74-1.99) |
| High-low § | 0.94 (0.54-1.63) | 1.03 (0.54-6.27) |  | 1.36 (0.73-2.55) | 1.34 (0.58-2.63) |  | 0.99 (0.55-1.79) | 1.06 (0.57-2.01) |
| LDL-C |  |  |  |  |  |  |  |  |
| Low-high ¶ | 0.98 (0.46-2.07) | 1.07 (0.45-2.57) |  | 0.81 (0.34-1.95) | 0.85 (0.33-2.02) |  | 0.38 (0.12-1.23) | 0.39 (0.12-1.27) |
| High-high ¶ | 1.08 (0.58-2.03) | 1.35 (0.60-3.11) |  | 0.81 (0.38-1.72) | 0.78 (0.35-1.49) |  | 1.06 (0.53-2.13) | 0.84 (0.43-1.82) |
| High-low § | 1.00 (0.50-1.99) | 1.04 (0.54-6.27) |  | 1.35 (0.60-3.05) | 1.33 (0.55-2.28) |  | 1.18 (0.56-2.50) | 1.39 (0.54-3.10) |
| HDL-C |  |  |  |  |  |  |  |  |
| Low-high ¶ | 1.27 (0.95-1.69) | 1.12 (0.81-1.56) |  | 1.05 (0.75-1.45) | 0.96 (0.68-1.37) |  | 1.08 (0.78-1.49) | 1.07 (0.78-1.49) |
| High-high ¶ | 1.31 (1.04-1.64) | 1.18 (0.90-1.54) |  | 1.32 (1.03-1.69) | 1.21 (0.90-1.62) |  | 1.24 (0.96-1.58) | 1.20 (0.91-1.57) |
| High-low § | 0.76 (0.55-1.06) | 0.76 (0.53-1.13) |  | 0.97 (0.67-1.36) | 1.00 (0.72-1.43) |  | 0.90 (0.63-1.28) | 0.91 (0.61-1.30) |

Model 1: unadjusted;

Model 2: adjusted for baseline age, education, marital status, smoking, drinking, BMI and exercise. Menstrual status was also adjusted in females.

¶ Take the low-low group as a reference; § Take the high-high group as a reference.

TC, total cholesterol; NHDL-C, non-high-density lipoprotein cholesterol; LDL-C, low-density lipoprotein cholesterol; HDL-C, high-density lipoprotein cholesterol.

**Supplemental Table 5** The OR (95% CI) between cholesterol variation and cognitive decline in participants with or without cardiovascular disease

| Cholesterol variation | Global cognitive decline |  | Memory function decline |  | Mental intactness decline |
| --- | --- | --- | --- | --- | --- |
|  | Model 1 |  | Model 1 |  | Model 1 |
| **At least one cardiovascular disease** | |  |  |  |  |
| TC |  |  |  |  |  |
| Low-high ¶ | 0.68 (0.35-1.33) |  | 0.70 (0.37-1.31) |  | 0.84 (0.43-1.66) |
| High-high ¶ | 1.06 (0.73-1.55) |  | 0.96 (0.66-1.39) |  | 1.02 (0.68-1.53) |
| High-low § | 0.64 (0.37-1.09) |  | 1.42 (0.88-2.29) |  | 0.79 (0.45-1.37) |
| NHDL-C |  |  |  |  |  |
| Low-high ¶ | 0.76 (0.36-1.63) |  | 0.53 (0.24-1.19) |  | 0.95 (0.44-2.03) |
| High-high ¶ | 1.03 (0.69-1.52) |  | 1.13 (0.78-1.63) |  | 0.95 (0.62-1.46) |
| High-low § | 0.66 (0.39-1.13) |  | 0.99 (0.62-1.59) |  | 0.75 (0.42-1.32) |
| LDL-C |  |  |  |  |  |
| Low-high ¶ | 1.10 (0.45-2.73) |  | 0.90 (0.36-2.23) |  | 0.83 (0.29-2.40) |
| High-high ¶ | 1.03 (0.62-1.72) |  | 0.91 (0.55-1.51) |  | 0.95 (0.55-1.65) |
| High-low § | 0.84 (0.46-1.54) |  | 1.38 (0.77-2.46) |  | 1.07 (0.56-2.05) |
| HDL-C |  |  |  |  |  |
| Low-high ¶ | 1.12 (0.83-1.52) |  | 1.01 (0.76-1.35) |  | 1.16 (0.85-1.58) |
| High-high ¶ | 1.26 (1.04-1.54) |  | 1.21 (1.01-1.47) |  | 1.08 (0.87-1.33) |
| High-low § | 0.83 (0.59-1.18) |  | 1.07 (0.78-1.47) |  | 0.99 (0.69-1.42) |
| **Without any cardiovascular disease** | |  |  |  |  |
| TC |  |  |  |  |  |
| Low-high ¶ | 0.63 (0.32-1.22) |  | 084 (0.48-1.49) |  | 0.78 (0.40-1.52) |
| High-high ¶ | 1.28 (0.88-1.86) |  | 0.99 (0.69-1.44) |  | 1.25 (0.84-1.87) |
| High-low § | 1.10 (0.66-1.82) |  | 1.46 (0.89-2.37) |  | 1.15 (0.67-1.96) |
| NHDL-C |  |  |  |  |  |
| Low-high ¶ | 0.37 (0.15-0.95) |  | 0.38 (0.16-0.89) |  | 0.91 (0.45-1.87) |
| High-high ¶ | 1.23 (0.83-1.81) |  | 1.16 (0.81-1.68) |  | 1.17 (0.77-1.79) |
| High-low § | 1.21 (0.72-2.04) |  | 1.19 (0.73-1.96) |  | 1.29 (0.74-2.26) |
| LDL-C |  |  |  |  |  |
| Low-high ¶ | 0.63 (0.25-1.63) |  | 0.78 (0.34-1.77) |  | 0.78 (0.30-1.99) |
| High-high ¶ | 1.20 (0.72-1.98) |  | 0.93 (0.56-1.53) |  | 1.12 (0.65-1.95) |
| High-low § | 1.10 (0.61-2.00) |  | 1.45 (0.80-2.60) |  | 0.95 (0.49-1.84) |
| HDL-C |  |  |  |  |  |
| Low-high ¶ | 1.13 (0.85-1.49) |  | 1.10 (0.85-1.43) |  | 0.90 (0.66-1.23) |
| High-high ¶ | 1.51 (1.23-1.84) |  | 1.24 (1.03-1.49) |  | 1.25 (1.02-1.55) |
| High-low § | 0.84 (0.63-1.11) |  | 0.89 (0.68-1.16) |  | 0.76 (0.55-1.05) |

Model 1: unadjusted;

¶ Take the low-low group as a reference; § Take the high-high group as a reference.

TC, total cholesterol; NHDL-C, non-high-density lipoprotein cholesterol; LDL-C, low-density lipoprotein cholesterol; HDL-C, high-density lipoprotein cholesterol.

**Supplemental Table 6** The OR (95% CI) between cholesterol variation and cognitive decline in participants just with hypertension and with heart problems or stroke

|  | Only with hypertension | | |  | With heart problems or stroke | | |
| --- | --- | --- | --- | --- | --- | --- | --- |
|  | Global cognitive  decline | Memory function  decline | Mental intactness  decline |  | Global cognitive  decline | Memory function  decline | Mental intactness  decline |
| TC |  |  |  |  |  |  |  |
| Low-high ¶ | 0.57 (0.24-1.36) | 0.68 (0.32-1.46) | 0.99 (0.47-2.10) |  | 0.55 (0.11-2.67) | 0.92 (0.25-3.38) | 0.23 (0.03-1.75) |
| High-high ¶ | 1.05 (0.55-1.99) | 0.75 (0.39-1.42) | 1.01 (0.53-1.91) |  | 0.76 (0.16-3.62) | 0.25 (0.03-2.00) | 1.62 (0.14-2.85) |
| High-low § | 0.48 (0.18-1.28) | 1.38 (0.55-5.37) | 0.61 (0.26-1.45) |  | 0.56 (0.23-2.28) | 1.46 (0.59-3.62) | 1.66 (0.19-14.24) |
| NHDL-C |  |  |  |  |  |  |  |
| Low-high ¶ | 0.86 (0.34-2.17) | 0.62 (0.25-1.54) | 0.84 (0.34-2.05) |  | 0.34 (0.04-3.04) | 0.38 (0.05-3.11) | 0.77 (0.16-3.70) |
| High-high ¶ | 1.00 (0.51-1.95) | 0.99 (0.53-1.83) | 1.16 (0.61-2.20) |  | 1.35 (0.33-5.46) | 1.10 (0.29-4.18) | 0.65 (0.14-2.98) |
| High-low § | 0.61 (0.24-1.55) | 1.40 (0.66-3.01) | 0.70 (0.30-1.61) |  | 0.87 (0.14-5.87) | 0.98 (0.15-6.25) | 1.06 (0.19-6.05) |
| LDL-C |  |  |  |  |  |  |  |
| Low-high ¶ | 0.95 (0.28-3.18) | 0.80 (0.26-2.49) | 0.89 (0.25-3.12) |  | 1.38 (0.20-9.46) | 1.45 (0.25-8.33) | 0.63 (0.08-5.25) |
| High-high ¶ | 0.94 (0.40-2.19) | 0.75 (0.33-1.71) | 0.48 (0.17-1.37) |  | 1.09 (0.21-5.75) | 0.37 (0.04-3.20) | 1.14 (0.22-5.83) |
| High-low § | 1.17 (0.43-3.17) | 1.96 (0.74-5.15) | 2.33 (0.69-7.77) |  | 1.01 (0.14-7.51) | 3.11 (0.33-6.12) | 0.39 (0.02-9.75) |
| HDL-C |  |  |  |  |  |  |  |
| Low-high ¶ | 1.06 (0.69-1.63) | 1.01 (0.69-1.47) | 1.11 (0.74-1.68) |  | 0.59 (0.28-1.23) | 0.72 (0.36-1.44) | 0.84 (0.44-1.59) |
| High-high ¶ | 1.18 (0.84-1.66) | 0.99 (0.73-1.35) | 1.17 (0.83-1.64) |  | 0.83 (0.48-1.45) | 1.15 (0.68-1.95) | 0.79 (0.45-1.39) |
| High-low § | 0.88 (0.54-1.44) | 1.16 (0.76-1.79) | 0.84 (0.52-1.38) |  | 0.62 (0.24-1.60) | 1.24 (0.59-2.59) | 1.21 (0.53-2.75) |

¶ Take the low–low group as a reference; §Take the high–high group as a reference. Baseline age, education, marital status, smoking, drinking, BMI, exercise, diabetes, history of disability, medication use (anti-hypertensive or anti-diabetic medications) and the number of comorbidity were adjusted. TC, total cholesterol; NHDL-C, non-high-density lipoprotein cholesterol; LDL-C, low-density lipoprotein cholesterol; HDL-C, high-density lipoprotein cholesterol; OR, odds ratio; CI, confidence interval.
